# Biflavonoids: Preliminary Reports on Their Role in Prostate and Breast Cancer Therapy

**DOI:** 10.3390/ph17070874

**Published:** 2024-07-02

**Authors:** Carolina Afonso de Lima, Larissa Kaori Maquedano, Luiza Sertek Jaalouk, Dina Cardoso dos Santos, Giovanna Barbarini Longato

**Affiliations:** Laboratory of Molecular Pharmacology and Bioactive Compounds, São Francisco University, 218 São Francisco Avenue, Bragança Paulista 12916-900, SP, Brazil; carolinaafonsolima@hotmail.com (C.A.d.L.); larissamaquedano8@gmail.com (L.K.M.); luiza.jaalouk@mail.usf.edu.br (L.S.J.); dina.santos@mail.usf.edu.br (D.C.d.S.)

**Keywords:** biflavonoids, breast and prostate cancer, anticancer, natural product

## Abstract

Dimeric flavonoids, also called biflavonoids, are bioactive compounds that exhibit various activities described in the literature, including antibacterial, antifungal, antiviral, anti-inflammatory, analgesic, antioxidant, vasorelaxant, and anticancer properties. This work focuses on the anticancer action of naturally occurring dimeric flavonoids against prostate and breast cancer, as well as on the mechanisms of action involved in their activity and presents the most current information on this subject in the literature. In the present review, we summarize the latest findings on the antiproliferative activity of 33 dimeric flavonoid-based compounds selected from recently published studies. The tests conducted were in silico and in vitro and demonstrated the cytotoxic activity potential of biflavonoids against prostate and breast tumor cells. Biflavonoids were capable of interfering with the migration and replication of cancer cells and their mechanism of action is related to cell death pathways, especially apoptosis, necrosis, and ferroptosis. These compounds decreased mitochondrial membrane potential and significantly increased intracellular levels of reactive oxygen species (ROS). Additionally, they significantly upregulated the expression of p21, Bax, and cleaved caspase-3, while downregulating Bcl-2 and caspase-3 levels, indicating their cell death mechanism of action is through the Bcl-2/Bax/cleaved caspase-3 pathway and cell cycle arrest. The biflavonoids here related have shown promising anticancer activity and are considered potential drug candidates for prostate and breast cancer treatment.

## 1. Introduction

According to the latest estimate published by the World Health Organization (WHO), in 2020, there were 1.4 million cases of prostate cancer worldwide, resulting in 397 thousand deaths from this disease and 19.3 million cases of breast cancer, resulting in 10 million deaths [1]. 

The incidence and mortality rates of prostate cancer worldwide are associated with increasing age, with the average age at the time of cancer diagnosis being around 66 years [2]. Breast cancer can be categorized into biologically and clinically significant subgroups based on histological grade. The histological grade, an assessment of the tumor’s proliferative activity (percentage of Ki67), reflects its aggressiveness and aids in determining the appropriate therapy [3]. 

Cancer treatment may involve surgery, chemotherapy, radiotherapy, and often requires a combination of modalities [4]. Despite advances in chemotherapy, many patients either do not opt for this intervention or must discontinue treatment, primarily due to side effects or the development of resistance to multiple drugs. This underscores the necessity and importance of researching new molecules that are more effective and have fewer adverse effects [5]. 

Natural products have long been used in the treatment of various diseases and are a crucial source for drug discovery research. One third of the chemotherapeutic drugs used in clinical cancer treatment originate from natural products or their derivatives [6]. Indeed, most molecules in clinical research, especially those targeting cancer and microbial infections, have been developed from natural products [7]. 

The natural products, particularly phytochemicals, have been the focus of extensive study. This is largely because they possess anticancer activities capable of interfering with the initiation, development, and progression of cancer by modulating several mechanisms, including cell proliferation, differentiation, apoptosis, angiogenesis, and metastasis [8]. These molecules generally have a higher molecular mass, a greater number of sp3 carbon and oxygen atoms, fewer nitrogen and halogen atoms, more hydrogen-bond acceptors and donors, a lower calculated octanol–water partition coefficient indicating higher hydrophilicity, and significantly greater molecular rigidity compared to synthetic compounds. These characteristics may prove beneficial in targeting protein–protein interactions in the drug discovery process [9].

Among natural compounds, flavonoids stand out as a broad group of secondary metabolites formed by polyphenolic compounds [10]. They exhibit diverse activities, including antibacterial, antiviral, anti-inflammatory, antioxidant, antiallergic, hepatoprotective, vasodilator, and antithrombotic effects. Additionally, they have the ability to inhibit cell proliferation, tumor growth, and carcinogenesis [11,12]. 

A new subclass of flavonoids, known as biflavonoids, has awakened scientific interest. Biflavonoids, composed of two monoflavonoid residues, occur naturally in angiosperms, bryophytes, ferns, and gymnosperms. They can be classified into three groups: C-C, C-linear fragment-C, or complex biflavonoids, depending on whether the linker between the two residues contains an atom. Since the linker can be established between two arbitrary rings from different residues, the C-C type encompasses various subtypes, as does the C-linear fragment-C type. [13]. Most members of this subclass consist of flavone–flavone and flavanone–flavanone dimers, with the dimers of chalcones and isoflavones occurring more rarely [14]. Biflavonoids have demonstrated a variety of biological activities, including anticancer, antibacterial, antifungal, antiviral, anti-inflammatory, analgesic, antioxidant, vasorelaxant, anticlotting [15], antibiotic, phospholipase A2 (PLA2), and cyclooxygenase-2 (COX-2) inhibition [16,17]. They are also involved in reversing multidrug resistance (MDR), and acting as mediators in the overexpression of resistance proteins such as MRP1 and P-glycoprotein (P-gp) in multidrug-resistant tumor cells [18]. 

Given the importance of natural products as sources of new medicines, and considering that prostate and breast cancer are the second most common cancer among the male and female population, respectively, and both present incidence rates that continue to increase every four years, there is a critical need to seek and investigate scientific data on the anticancer properties of biflavonoids on these specific types of cancer.

## 2. Results

Twenty-eight articles related to the anticancer properties of dimeric flavonoids against prostate (Table 1) and breast (Table 2) cancer cells were found. 

**Figure 1:**
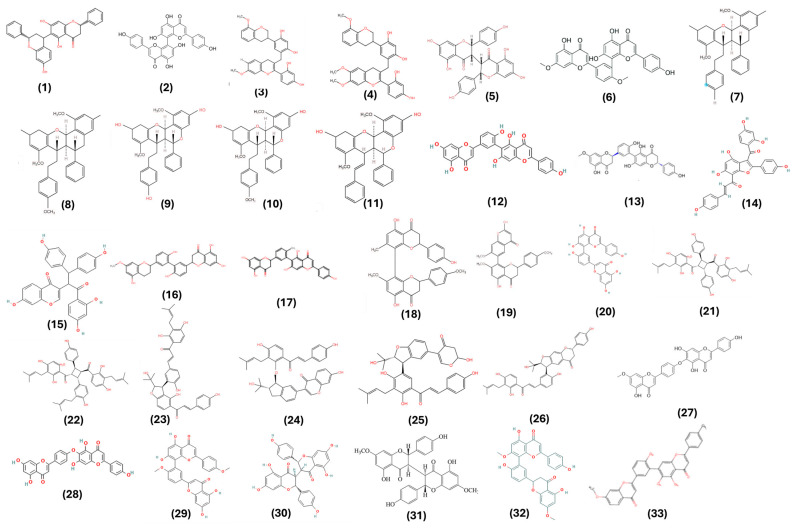
Molecular structure of biflavonoids **1**–**33**.

All of these studies employed in vitro and in silico tests, with the MTT assay being the most commonly selected method to evaluate the cell viability of both tumor types. Tests involving the ability of these compounds in inhibiting the migration and clonogenicity were also executed. Furthermore, genes and proteins associated with apoptosis pathways were investigated. The prostate cancer cell lines PC-3, DU145, LNCaP, and non-tumoral PNT2 and the breast cancer cell lines MCF7 and MDA-MB-123 were used in these tests. Thirty-three dimeric flavonoids, numbered 1 to 33 (Figure 1), were identified and their anticancer activities were reported below, in accordance with their findings. The biflavonoids were categorized by subtype (C-C, C-linear fragments-C, and complex biflavonoids) and monomer type, including: AA (flavan–flavan), BB (flavone–flavone), EE (isoflavone–isoflavone), EG (isoflavone–chalcone), and GG (chalcone–chalcone) [13]. 

Oxitrodiflavanone A (**1**) is a biflavonoid isolated from *Oxytropis chiliophylla*. Its cytotoxic activity against prostate tumor cells (PC-3) was evaluated using the 3-(4,5-dimethylthiazol-2-yl)-2,5-diphenyltetrazolium bromide (MTT) cell viability assay. The IC50 value obtained was 6.64 µM, indicating that the biflavonoid exhibits potential cytotoxic activity against the PC-3 cell line [19]. 

The biflavonoid Cupressoflavone (**2**) was isolated from ground leaves of *Juniperus phoenicea* L. This compound has been previously tested and demonstrated anticancer, antioxidant, antimicrobial, antinociceptive, and anti-inflammatory activities. Its cytotoxic effect was evaluated against the prostate cancer cell line PC-3 and the non-tumor prostate cell line PNT2 using the MTT assay. Cupressoflavone showed high cytotoxic selectivity for prostate cancer cells (PC-3) with an IC50 value of 19.9 μM, while showing no cytotoxicity against the normal prostate cell line (PNT2). This selectivity for prostate tumor cells is noteworthy. The result is particularly significant when compared to etoposide, a commonly used anticancer drug, which had an IC50 value of 61 μM—three times higher than that of Cupressoflavone [14,21].

Red propolis is derived from plant sources and contains various compounds that can be isolated, including two dimeric flavonoids named Propolone B (**3**) and Propolone A (**4**). These compounds demonstrated antiproliferative effects in the prostate cancer cell line (PC-3) using the MTT assay, with Propolone B showing a TGI (total growth inhibition) of 19.1 µM and Propolone A showing a TGI of 21.9 µM. This highlights their potential antiproliferative effects in 2D in vitro cultures [21].

A biflavonoid isolated from the roots of traditional Chinese medicine, *Stellera chamaejasme* L., named Neochamaejasmin A (**5**), was analyzed in a prostate cancer cell line (LNCaP). The IC50 value obtained was 12.5 μg/mL. Treatment with low concentrations (≤6.25 μg/mL) of compound 5 inhibited the expression of cell cycle regulatory proteins such as cyclin D and the cyclin-dependent kinase inhibitor p21, leading to cell cycle arrest in the G1 phase. Additionally, this biflavonoid altered mitochondrial membrane potential and induced cellular apoptosis in LNCaP cells through the Fas-caspase8-caspase3 pathway [22].

Ginkgetin (**6**) is a natural biflavonoid isolated from the leaves of *Ginkgo biloba* L. This molecule exhibits cytotoxic effects on prostate cancer cell lines PC-3 and DU-145, with IC50 values of 15 μM and 5 μM, respectively. It induces cell cycle arrest in the sub-G1 phase and activates apoptosis through caspase-3 and PARP cleavage [23,24].

Furthermore, Ginkgetin inhibits tumor growth as demonstrated in a mouse xenograft model using DU-145 cells, highlighting its potential as a potent anticancer agent suitable for clinical use. Ongoing studies by the research group aim to further elucidate the compound’s mechanism of action [24].

Brachydins (Br) are biflavonoids extracted from the roots of *Fridericia platyphylla* (Cham.) L.G. Lohmann. They have recently been extensively studied by several research groups, and the results concerning their effects on prostate and breast cancer cells are presented here. Brachydin E (**7**) and Brachydin F (**8**) significantly reduced the cell viability of the prostate cancer cell line PC3, with IC50 values of 6.9 μM and 37.1 μM, respectively. In addition, wound healing and clonogenicity assays were conducted. Both biflavonoids effectively inhibited cell repopulation, resulting in only 20% and 13.2% closure of the wound area after 48 h of treatment, respectively. Furthermore, they reduced the number of PC-3 cell colonies by more than 60%. 

The cell death assay assessed phosphatidylserine externalization in PC3 cells using Annexin-V labeling. Brachydin E (**7**) and Brachydin F (**8**) induced an increase in Annexin V-labeled cells, indicating their ability to induce regulated cell death in prostate tumor cells. Additionally, an in silico study demonstrated that Brachydins E and F target nuclear receptors, specifically molecularly coupling with the glucocorticoid receptor (GR). Glucocorticoid hormones (GC) are known to exert an antiproliferative effect on various cells through the GR, which acts as a transcription factor. Thus, the direct binding of these compounds to the glucocorticoid receptor contributes to their antiproliferative function in cancer cells [25].

The Brachydins (Br) A (**9**), B (**10**), and C (**11**) were evaluated for their cytotoxicity against the prostate cancer cell line (PC-3) using the MTT assay, yielding IC50 values of 23.41 µM (**9**), 4.28 µM (**10**), and 4.44 µM (**11**). They were further assessed by the neutral red cytotoxicity assay, revealing that compounds **9**, **10**, and **11** initiated cytotoxic effects at concentrations of 15.36 µM, 6 µM, and 3.84 µM, respectively. At these concentrations, these biflavonoids induced cell death in the PC-3 cell line, as confirmed by the LDH activity release assay. The cell death mechanisms of Brachydins were investigated, showing that BrA and BrC induce necrosis in cells, while BrB can induce both necrosis and apoptosis in prostate cancer cells. Protein analysis via Western blot revealed overexpression of the p21 protein, leading to cell cycle arrest in PC-3 cells treated with BrB or BrC, but not BrA. The p27 protein, another cell cycle regulator, remained unchanged with the treatments. Expression of pAKT was reduced in cells treated with BrA and BrB, indicating disruption of cell survival processes. Additionally, cleaved PARP expression was elevated across all treatments, suggesting cell death via apoptosis. These findings underscore the potent antiproliferative effects of these biflavonoids, although further studies are needed to fully elucidate their mechanisms of action [26].

The antiproliferative and antimetastatic activity of Brachydin A (**9**) was evaluated in a three-dimensional (3D) culture of DU145 prostate cancer cells. Brachydin A exhibited cytotoxic effects at concentrations ranging from 60 to 100 μM, leading to alterations in spheroid morphology and volume, as well as suppression of cell migration and tumor invasiveness. In addition, Brachydin A caused a reduction in mitochondrial membrane potential, resulting in increased markers of apoptosis and necrosis, including activation of cleaved PARP and p-γ-H2AX. It also decreased levels of anti-apoptotic/pro-apoptotic markers such as BCL-2, BAD, and RIP3K, as well as cell survival markers p-AKT1 and p-44/42 MAPK. There was an elevation in the protein levels of effector caspases (CASP3, CASP7, and CASP8) and a positive regulation of inflammation markers (NF-kB and TNF-α). These findings highlight Brachydin A as a potential candidate for preclinical studies against metastatic prostate cancer due to its multifaceted effects on cell viability, apoptosis, necrosis, and inflammation [27].

Brachydin B (**10**) was evaluated in both two-dimensional (2D) and three-dimensional (3D) cultures of the prostate cancer metastatic cell line DU145. It was found to induce cytotoxic effects within 24 h at a concentration of 7.45 μM in 2D culture, whereas in 3D culture, cytotoxic effects were observed at concentrations above 50 μM within 48 h. Brachydin B reduced clonogenicity in 2D culture and decreased the area/volume of 3D spheroids. It also demonstrated the ability to inhibit cell migration and invasion in both 2D and 3D assays, highlighting its potential as a potent anticancer agent in vitro. Further in vivo studies are planned to confirm its candidacy for therapy against metastatic prostate cancer [28].

Brachydin C (**11**) was also investigated in prostate cancer cells (DU145) using both 2D and 3D culture models. The IC50 values after 24 h of treatment were determined to be 47.31 μM (2D) and 229.8 μM (3D). Brachydin C impaired both horizontal (wound healing) and vertical (transwell assay) cell migration and invasion in 2D culture. Additionally, Brachydin C modulated the expression of several genes including BIRC5, TNF-α, CASP3, NKX3.1, MMP9, MMP11, CDH1, and ITGAM. In Western blot analysis, it downregulated proteins such as CASP7, BAX, and TNF-α. Overall, Brachydin C induced cell death and affected epithelial–mesenchymal transition processes [29].

The cytotoxic effect of a dichloromethane fraction (DCMF) containing the three brachydins (**9**–**11**) was evaluated on the prostate cancer cell line DU145 and the non-tumoral prostate cell line PNT2. The IC50 value for the prostate cancer cells was found to be half the concentration observed in the non-tumoral cell line, indicating the selectivity of DCMF towards cancer cells. These compounds together significantly inhibited colony formation and reduced migration of prostate tumor cells. Atomic force microscopy (AFM) was employed to examine changes in the cell membrane, revealing an increase in the number of membrane holes and roughness, particularly in tumor cells, in a concentration-dependent manner. These findings corroborate the observed effects of cytotoxicity, inhibition of clonogenicity, and suppression of migration in cells treated with DCMF [30].

The dichloromethane fraction (DCMF) containing Brachydin A (**9**), B (**10**), and C (**11**) was also analyzed for breast cancer, through the MCF7 lineage. It was observed that DCMF reduces cell viability, with IC50 values of 2.77 µg/mL for the strain under study. At higher concentrations, they were able to significantly inhibit the migration of cell lines and altered the membrane structures of tumor cells without causing toxic effects to normal cells [30].

The cytotoxic effect of the biflavonoid Robustaflavone (**12**), isolated from a species of *Selaginella* called *S. trichoclada*, on MCF7 breast cancer cells, was evaluated through MTT assay and the IC50 found was 11.89 μΜ. Subsequent transcriptome analysis identified VDAC2 (voltage-dependent anion selective channel protein 2) as a potential target of compound **12**, a key regulator in the ferroptosis cell death pathway. These findings suggest that compound **12** may induce MCF7 cell death through non-apoptotic pathways, characterized by diminished or absent mitochondrial cristae, a hallmark of ferroptosis due to the accumulation of reactive oxygen species, making it an interesting study, given that the vast majority of dimeric flavonoids do not act through cell death by ferroptosis [31]. 

The antiproliferative activity of a new biflavonoid called (2R,2′R′)-7-O-methyl-2,3,2″,3″-tetrahydrorobustaflavone (**13**), isolated from *Aster tataricus*, whose structure was confirmed through spectroscopic and circular dichroism analysis, was evaluated against seven human cancer cell lines: A549 (lung), NCI-H1975 (lung), PC3 (prostate), DU145 (prostate), HepG2 (liver), LoVo (colon) and MCF-7 (breast) using the MTT assay. The compound under study showed cytotoxicity (IC50 = 5.4 μM) exclusively against MCF-7 cells [32]. 

Two biflavonoids isolated from the same plant species *Brackenridgea zanguebarica*, Calodenin B (**14**) and Lophirona A (**15**), were analyzed for their cytotoxic activity against MCF7 breast tumor cells, and their EC50 values were 19.2 μM for Lophirone A and 219.3 μM for Calodenin B, indicating that Lophirone A is cytotoxic to MCF7 breast cancer cells, while Calodenin B is not effective [33] and this may be attributed to their chemical structure. 

Other biflavonoids with cytotoxic activities were discovered in *Selaginella doeder-leinii*, highlighting their potential as plant-derived anticancer agents, called 7″-O-methylrobustaflavone (**16**) and 4‘-O-methylrobustaflavone (**17**). The cytotoxic activity of these compounds was tested against four human tumor cell lines: A549 (lung), MCF7 (breast), SMMC7721 (liver) and LoVo (colon) using the MTT assay. The results revealed that 7″-O-methylrobustaflavone (**16**) exhibited selective modest activity for MCF7 cells with an IC50 value of 15.09 µM, and 4′-O-methylrobustaflavone (**17**) showed moderate inhibitory effects in three tumor cell lines (MCF7, SMMC-7721, and LoVo) with IC50 values ranging from 16.68 to 33.47 µM [34].

The compounds 4′,7,7″-tri-O-methylcuppressuflavone (**18**) and 4″,7,7″-tri-O-methylagathisflavone (**19**) were initially isolated from the leaves of the Indonesian *A. hun-steinii* and later from other Araucaria plants. An MTT assay on MCF7 breast cancer cells revealed IC50 values of 91.74 µg/mL for compound **18** and 314.44 µg/mL for compound **19**, showing that compound **18** can be active against MCF7 cancer cells, while compound **19** is not, although both presented IC50 values higher than the positive control epirubicin HCl (IC50 0.52 µg/mL) [35].

The cytotoxicity of the flavonoids Amentoflavone (**20**) and Cupressoflavone (**2**), derived from the active fractions (F3 and F4) of the methanolic extract of *J. phoenicea* leaves, was investigated by Groshi (2019). Among all human cancer cell lines evaluated, MCF7 and MDA-MB-231 were most sensitive to compounds **20** and **2**, with IC50 values of 25 μM for MCF7 and 12.7 μM for MDA-MB-231, indicating specific cytotoxicity. In relation to breast cancer cell lines, their mechanisms will be studied for better analysis, as they are two different types of breast tumors and with different receptors [20].

Nine new biflavonoids were isolated from the fruits of *Psoralea corylifolia*. In MTT assays, compounds **21**, **23**, **25,** and **26** demonstrated cytotoxicity with IC50 values of 7.35, 10.01, 8.42, and 9.01 μM, respectively, while compounds **22** and **24** showed mild cytotoxic activities, with IC50 values of 17.40 and 21.98 μM, respectively. Compounds **21** and **26** were notable for their antiproliferative activity against breast cancer cells, as they led to a decrease in mitochondrial membrane potential, significantly increased intracellular reactive oxygen species (ROS) levels, and induced apoptosis in MCF7 cells. Specifically, compound **26** exhibited an apoptosis ratio of 6.17 ± 0.39% at low concentration (4 μM) with a significant decrease in mitochondrial membrane potential, and compound **21** showed an apoptosis ratio of 24.58 ± 1.68% at 8 µM. Furthermore, they upregulated the expression of Bax and cleaved caspase-3, while downregulated the levels of Bcl-2 and caspase-3, indicating their action through the Bcl-2/Bax/cleaved caspase-3 pathway. These biflavonoids have demonstrated promising anticancer activity and are considered potential drug candidates for the treatment of breast cancer [36].

The biflavonoids cryptomerin (**27**) and hinokiflavone (**28**) were isolated from *Selaginella tamariscina* (Beauv.). Initial trials revealed IC50 values of 30.09 µg/mL for cryptomerin (**27**) and 39.32 µg/mL for hinokiflavone (**28**) against the breast cancer cell line MCF7. These findings indicate cytotoxic effects of these biflavonoids against breast cancer cells. Further studies are required to fully explore and characterize their potential anticancer properties [37].

Hinokiflavone (**28**) is a biflavonoid isolated from *Selaginella p*. Beauv, *Juniperus phoenicea*, and *Rhus succedanea*. Known for its various biological activities such as anti-HIV-1, reverse transcriptase inhibition, anti-sialic acid influenza enzyme, and antioxidant properties, its anticancer activity against MDA-MB-231 breast tumor cells has been thoroughly investigated both in vitro and in vivo. In vitro studies demonstrated that hinokiflavone induced apoptosis in MDA-MB-231 cells at a concentration of 40 μM. Additionally, it exhibited significant anti-migration and anti-invasion effects in a dose-dependent manner. In vivo evaluation using a mouse xenograft model of MDA-MB-231 tumors showed that hinokiflavone treatment over 21 days significantly inhibited tumor growth at doses of 20 mg/kg and 40 mg/kg. Tumor weight reduction was observed in a dose-dependent manner, and immunohistochemical staining revealed fewer Ki67-positive and MMP-2-positive cells in tumors treated with hinokiflavone compared to the control group. These findings underscore hinokiflavone’s ability to suppress the growth of human breast cancer cells in vivo, supporting the results obtained from in vitro studies [38].

Isoginkgetin (**29**), a biflavonoid extracted from the leaves of *M. glyptostroboides* (Dawn redwood, family: Taxodiaceae), has demonstrated potent antitumorigenic properties. In studies conducted on MDA-MB-231 breast cancer cells, Isoginkgetin was found to reduce the production of matrix metalloproteinase MMP-9, Akt, and PI3K. These proteins play crucial roles in cancer progression, particularly in invasion processes. Given that Isoginkgetin effectively decreases these proteins implicated in invasion, it emerges as a promising candidate for therapeutic intervention against tumor invasion of MDA-MB-231 cells [39].

Chamaejasmin (**30**), a new biflavonoid derived from *Stellera chamaejasme* L., exhibited an IC50 value of 4.72 μM against MDA-MB-231 breast cancer cells. It induced cell cycle arrest in the G2/M phase by inhibiting cyclins Cdk2 and cdc2, and activating WAF1/p21 and KIP1/p27. Additionally, Chamaejasmin induced apoptosis by activating Bax and inhibiting Bcl-2. These findings highlight Chamaejasmin as a potent anticancer agent that acts on multiple cell death pathways, inhibiting the growth of MDA-MB-231 cells [40].

7,7″-di-O-methylchamaejasmin (**31**), isolated from the Kenyan medicinal plant *Ormocarpum kirkii*, demonstrated an IC50 value of 7.76 μM against MDA-MB-231 breast cancer cells. It induced apoptosis by altering mitochondrial membrane potential and increasing reactive oxygen species [41].

Two biflavonoids, Podocarpusflavone-A (**32**) and II-4″,I-7-Dimethoxyamentoflavone (**33**), were isolated from the dry twigs of *Podocarpus nakaii* Hayata (Podocarpaceae). Their cytotoxic activity was evaluated in the breast cancer cell line MCF7, revealing ED50 values of 16.24 µg/mL and 15.17 µg/mL, respectively. Mechanistic studies indicated that both biflavonoids induce cell cycle arrest at the S phase in MCF7 cells, which was associated with alterations in Topoisomerase I enzyme activity [42].

Ginkgetin (**6**), previously mentioned, was also evaluated in MCF7 breast cancer cells, where it exhibited an IC50 value of 10 µM. It increased the number of cells labeled with annexin V and PI, indicating cell death by apoptosis. Ginkgetin also inhibited the estrogen receptor signaling pathway by downregulating ER-α expression. This explains the lack of promising results in the MDA-MB-231 cell line, which lacks estrogen receptors [44].

The biflavonoid amentoflavone (**20**), previously mentioned, was also assessed in MCF7 breast cancer cells, revealing an IC50 of 150 μM. It induced cell cycle arrest in the G1 phase and promoted chromatin condensation and apoptosis. The comet assay demonstrated that amentoflavone induced DNA damage. Protein analysis indicated a decrease in BCL2 levels and an upregulation of BAX in MCF7 cells, underscoring its significance for clinical research [43].

## 3. Discussion

In recent decades, special attention has been given to dimeric flavonoids, which are prevalent in various species of the plant kingdom. Although well reported for their rich pharmacological properties in the literature, there remains a scarcity of articles on their anticancer activity [16]. 

The results regarding the anticancer potential of the dimeric flavonoids discussed here align with findings in the literature on the anticancer activity of this subclass of flavonoids in other cancer types, such as glioblastoma, lung, and neuronal cell lines. Most of the trials described herein are preliminary, indicating that research in this area is still in its early stages and merits further attention from the scientific community.

This review presented the results of 33 dimeric flavonoids on the antiproliferative activity against prostate and breast cancer. Generally, the tests carried out with these compounds were in vitro, with the majority demonstrating cytotoxicity through the MTT assay. The lowest IC50 values found for prostate and breast cancer cells were obtained with brachydins derived from the genus *Fridericia platyphylla* (Cham.) L.G. Lohmann. These molecules are classified as monomer type G-G, resulting from the union of two chalcones. The presence of chalcones in the structure of Brachydins contributes significant pharmacological potential, as this group of compounds exhibits diverse properties, including antioxidant, cytotoxic, anticancer, antimicrobial, antiprotozoal, antiulcer, antihistamine, and anti-inflammatory activities. Various pharmacologically active compounds have been developed based on the chalcone skeleton [25]. 

Biflavonoids were capable of interfering with the migration and replication of cancer cells, on the cell cycle progression and their mechanism of action is related to cell death pathways, specially apoptosis, necrosis and ferroptosis. Apoptosis is a disordered cellular process and also one of the most studied regulated deaths by scientists. In cancer, this process occurs in a minimal way, allowing cells to survive for longer and to multiply progressively, allowing the transformation of cancer cells, tumor metastasis and developing resistance to multiple drugs, therefore compounds that can induce apoptosis in tumor cells play an important role in the treatment of cancer and are a target of many treatment strategies [45]. The term necrosis refers to a series of events, such as a gain in cell volume, swelling of organelles, rupture of the plasma membrane with consequent loss of intracellular content. In the same way as apoptosis, it is considered a regulated cell death, and also a type of cell death for the treatment of cancer, as it causes the total extravasation of the contents of that cancer cell treated [46]. Ferroptosis is a form of regulated cell death specific to iron overload, accumulation of lipid reactive oxygen species (ROS) and lipid peroxidation. Evidence demonstrates that this type of cell death is directly linked to cancer suppression [47].

Therapy that targets cancer also acts by interrupting the functions of proteins that play essential roles during cancer progression. Biflavonoids were shown to be positive in inhibiting proteins that induce tumor growth (oncoproteins), as well as activating proteins directly related to regulated cell death (tumor suppressor proteins). We can also highlight that dimeric flavonoids have selectivity for tumor cells and presented, in vitro, less toxicity than other chemotherapeutic drugs still used clinically. Only two articles have investigated the in vivo anticancer effects of the bioflavonoids ginkgetin (**6**) and hinokiflavone (**28**). Pre-clinical tests involving toxicity still need to be conducted [48]. 

The most studied cell lines were PC-3 (prostate) and MCF7 (breast), likely because these tumor types are more responsive to existing treatments and are commonly used in in vitro research. Additionally, some research groups explored the effects of biflavonoids on the MDA-MB-231 breast cancer cell line, derived from a triple-negative strain. The findings related to compounds **2**, **20**, and **28**–**31** are particularly noteworthy since triple-negative breast cancer lacks estrogen and progesterone receptors and has insufficient HER2 protein, making it ineligible for hormone therapy or HER2-targeted medications, thus limiting treatment options compared to other types of invasive medications for breast cancer.

The research on biflavonoid anticancer properties is still emerging in the literature, indicated by the limited number of articles published over a period of six years. This scarcity can be attributed to biflavonoids because it is only recently that they have gained significant scientific interest as potent bioactive compounds. We strongly encourage further research into the anticancer properties of biflavonoids, as they presented promising results and are candidates for new anticancer agents.

Cancer is considered a global problem, due to its high rate of occurrence and mortality, and unfortunately the treatments that exist present many adverse effects. Considering that prostate and breast cancer are the most recurrent in the population, the option of new treatments is necessary, as a way to have fewer adverse effects and a high rate of selectivity for tumor cells. In this sense, biflavonoids have high selectivity for tumor cells and low adverse effects, so studies need to move on to the next phases of development to better explore the mechanism of action by which they act on these certain types of cancer and obtain new compounds for the treatment of prostate and breast cancer. We strongly encourage further in vivo experiments with biflavonoids. 

## 4. Materials and Methods

This study was carried out through a literature review, using the PubMed and Sci-ELO platforms to search for articles published in recent years, with the keywords “dimers of flavonoids”, “flavonoid dimers”, “biflavonoids”, “dimeric flavonoids” isolated or together with “prostate cancer” or “breast cancer”. The articles selected for this review were those categorized as studies on naturally occurring flavonoid dimers, with their structures explicitly stated. Only original research articles were considered.

## Figures and Tables

**Table 1:** List of biflavonoids that have exhibited cytotoxic effects on prostate cancer cells.

Biflavonoids	Subtype	Monomer Type	Cell Lines	Assays	*IC50/#EC50/@TGI	Authors
Oxitrodiflavanone A (**1**)	C-C	BB	PC-3	MTT (cell viability)	6.64 μΜ (**1**)	[19]
Cupressuflavone (**2**)	C-C	AA	PC-3	MTT (cell viability)	19.9 μΜ (**2**)	[20]
Propolone B (**3**) Propolone A (**4**)	C-linear fragment-C	EE	PC-3	MTT (cell viability)	19.1 μM (**3**) 21.9 μM (**4**)	[21]
Neochamaejasmin A (**5**)	C-C	AA	LNCaP	MTT (cell viability) Western blot analysis Cell cycle analysis	12.5 μg/mL (**5**)	[22]
Ginkgetin (**6**)	C-C	BB	PC-3	MTT (cell viability) Western blot analysis Cell cycle analysis	15–30 μM (**6**)	[23]
Ginkgetin (**6**)	C-C	BB	DU-145	MTT (cell viability) Western blot analysis Cell cycle analysis Mouse xenograft in vivo	5 μM (**6**)	[24]
Brachydin E (**7**) Brachydin F (**8**)	Complex	GG	PC-3	MTT (cell viability), Molecular docking, Wound healing assay, Clonogenic assay, Phosphatidylserine (PS) Externalization assay, In silico pharmacodynamics	6.9 μM (**7**) 37.1 μM (**8**)	[25]
Brachydin A (**9**) Brachydin B (**10**) Brachydin C (**11**)	Complex	GG	PC-3	MTT (cell viability) Neutral red assay, LDH activity release assay, Cell death assay, Comet assay, Western blot analysis	23.41 μM (**9**) 4.28 μM (**10**) 4.44 μM (**11**)	[26]
Brachydin A (**9**)	Complex	GG	DU145	Cytotoxicity assay, Tumor spheroids, Clonogenicity, Cell migration, Cell death assay, Protein Expression	60.0–100.0 μM (**9**)	[27]
Brachydin B (**10**)	Complex	GG	DU145	MTT (cell viability), Clonogenicity, Cell death assay, LDH, Cell migration	7.45 μM (**10**)	[28]
Brachydin C (**11**)	Complex	GG	DU145	Cytotoxicity assay, Cell migration, Clonogenicity, Protein expression	47.31 μM (**11**)	[29]
DCMF containing Brachydin A (**9**) Brachydin B (**10**) Brachydin C (**11**)	Complex	GG	DU145	Sulforhodamine B (cell viability), Comet (genotoxicity), Clonogenicity (reproductive capacity) and Wound healing (cell migration) assays, and Atomic force microscopy (AFM) for ultrastructural cell membrane alterations	2.51 μg/mL (**9**–**11**)	[30]

*IC50—Half-maximal inhibitory concentration. #EC50—Half-maximal effective concentration. @TGI—Total growth inhibition concentration.

**Table 2:** List of biflavonoids that presented cytotoxic effects on breast cancer cells.

Biflavonoids	Subtype	Monomer Type	Cell Lines	Assays	*IC50/#EC50/@TGI	Authors
DCMF containing Brachydin A (**9**) Brachydin B (**10**) Brachydin C (**11**)	Complex	GG	MCF7	Sulforhodamine B (cell viability), Comet (genotoxicity), Clonogenicity (reproductive capacity), Wound healing (cell migration) assays, and Atomic force microscopy (AFM) for ultrastructural cell membrane alterations	2.77 μg/mL (**9**–**11**)	[30]
Robustaflavone (**12**)	C-C	BB	MCF7	MTT (cell viability), Detection of apoptotic cells, RNA extraction and sequencing, ROS, Molecular docking, Western blot	11.89 μΜ (**12**)	[31]
(2R,2′R′)-7-O-methyl-2,3,2″,3″-tetrahydrorobustaflavone (**13**)	C-C	AA	MCF7	MTT (cell viability)	5.4 μΜ (**13**)	[32]
Calodenin B (**14**) Lophirone A (**15**)	C-C (**14**) Complex (**15**)	AA (**14**) EG (**15**)	MCF7	Cytotoxicity assay	219.3 μM (**14**) 19.2 μM (**15**)	[33]
7-O-methyl-2,3,2″,3″-tetrahydro-3′,3‴-biapigenin (**16**) 4′-O-methylrobustaflavone (**17**)	C-C	BB	MCF7	MTT (cell viability)	41.44 μM (**16**) 16.68 μM (**17**)	[34]
4′,7,7″-tri-O-methylcupressuflavone (**18**) 4‴,7,7″-tri-O-methylagathisflavone (**19**)	C-C	BB	MCF7	MTT (cell viability)	91.74 μg/mL (**18**) 314.44 μg/mL (**19**)	[35]
Amentoflavone (**20**) Cupressuflavone (**2**)	C-C	AA	MDA-MB-231	MTT (cell viability)	16.1 μM (**20**) 12.7 μM (**2**)	[20]
Psocorylin R (**21**) Psocorylin S (**22**) Psocorylin U (**23**) Psocorylin V (**24**) Psocorylin W (**25**) Psocorylin Y (**26**)	C-C or C-linear fragment-C	GG	MCF7	Cytotoxicity assay and Apoptosis assay	7.35 μM (**21**) 17.40 μM (**22**) 10.01μM (**23**) 21.98 μM (**24**) 8.42 μM (**25**) 9.01 μM (**26**)	[36]
Neocryptomerin (**27**) Hinokiflavone (**28**)	C-linear fragment	BB	MCF7	MTT (cell viability)	30.09 µg/mL (**27**) 39.32 µg/mL (**28**)	[37]
Hinokiflavone (**28**)	C-linear fragment	BB	MDA-MB-231	MTT (cell viability), Clonogenicity (reproductive capacity), Western blot, cell migration, In vivo tumor model in mice	40 μM (**28**)	[38]
Isoginkgetin (**29**)	C-C	BB	MDA-MB-231	Cytotoxicity assay Western blot	20 μM (**29**)	[39]
Chamaejasmin (**30**)	C-C	AA	MDA-MB-231	MTT (cell viability), Western blot, Cell cycle analysis	5.11 μM (**30**)	[40]
7,7″-di-O-methylchamaejasmin (**31**)	C-C	AA	MDA-MB-231	Cytotoxicity assay, Western blot, Apoptosis assay	7.76 μM (**31**)	[41]
Podocarpusflavone-A (**32**) II-4″,I-7-Dimethoxyamentoflavone (**33**)	C-C	BB	MCF7	MTT (cell viability), Cell cycle Topoisomerase I assay	16.24 µg/mL (**32**) 15.17 µg/mL (**33**)	[42]
Amentoflavone (**20**)	C-C	AA	MCF7	MTT (cell viability), Cell cycle Western blot Cometa assay	150 μM (**20**)	[43]
Ginkgetin (**6**)	C-C	BB	MCF7	MTT (cell viability), Cell cycle Western blot Apoptosis assay ER-α expression	10 µM (**6**)	[44]

*IC50—Half-maximal inhibitory concentration. #EC50—Half-maximal effective concentration. @TGI—Total growth inhibition concentration.

## Data Availability

The data presented in this study are available on request from the corresponding author due to privacy restriction.
